# Effects of *Bellamya purificata* Cultivation at Different Stocking Densities on the Dynamics and Assembly of Bacterial Communities in Sediment

**DOI:** 10.3390/biom13020254

**Published:** 2023-01-30

**Authors:** Mengmeng Zhou, Yiran Hou, Rui Jia, Bing Li, Jian Zhu

**Affiliations:** 1 College of Fisheries and Life Science, Shanghai Ocean University, Shanghai 201306, China; 2 Key Laboratory of Integrated Rice-Fish Farming Ecology, Ministry of Agriculture and Rural Affairs, Freshwater Fisheries Research Center, Chinese Academy of Fishery Sciences, Wuxi 214081, China

**Keywords:** *Bellamya purificata*, aquaculture, bacterial community assembly, sediment, stocking density

## Abstract

To optimize the integrated multi-trophic aquaculture (IMTA) model, improve the efficiency of resource utilization, and reduce environmental pollution, *Bellamya purificata,* as a potential bioremediation species, was studied to investigate the effect of *B. purificata* culture on the dynamics and assembly of bacterial communities in sediment. Four experimental groups were established at four different densities: 0, 234.38, 468.75, and 937.5 g/m^2^ (represented as CON, LD, MD, and HD, respectively). Each group was with three replicates. The 16S ribosomal RNA (rRNA) high-throughput sequencing was used to evaluate the composition, function, and assembly of bacterial communities in sediment. *B. purificata* cultivation significantly altered the composition and function of the bacterial communities in sediment; at high stocking density, it significantly decreased anaerobic and increased aerobic organic matter decomposition, whereas at low stocking density, it decreased the number of bacteria involved in sulfate reduction and inhibited the denitrification process. *B. purificata* decreased direct competition and promoted collaboration or niche sharing in bacterial communities, especially at the high stocking density. Moreover, *B. purificata* cultivation resulted in greater changes in the environmental factors. Variations in dissolved oxygen, pH, total nitrogen, nitrate, and nitrite levels were closely related to the altered composition and function of the bacterial communities. Stochastic processes dominated the bacterial community assembly in the sediment and *B. purificata* cultivation had limited impacts on the bacterial community assembly. The study provided a reference for the dynamics and assembly of bacterial communities in sediment with different densities of *B. purificata* cultivation and we hope that the findings will provide a theoretical reference for the optimization of IMTA and improve management strategies for *B. purificata* polyculture.

## 1. Introduction

Aquaculture plays a vital role in food security and meeting the increasing global demand for nutritious food [1]. In 2020, global aquaculture production reached a record 122.60 million tons, worth USD 281.5 billion [2]. China’s total aquaculture production ranked first in the world with 52.24 million tons in 2020; approximately 24.81 million tons were farmed in aquaculture ponds [3]. With the rapid development of aquaculture and its increasing contribution to food safety and security, aquaculture ecosystems including farming ponds, lakes, reservoirs, and rivers, have become increasingly important. Aquaculture is the breeding, cultivation, and harvesting of aquatic products under artificial feeding management. However, traditional aquaculture focused on economic efficiency and production but too high a stocking density and excessive feed input in aquaculture ecosystems have led to environmental stress and other serious issues.

To solve these problems, integrated multitrophic aquaculture (IMTA) has been studied and applied in actual culture processes. IMTA is a sustainable farming method that is currently being vigorously developed; it mainly consists of a combination of multiple culture units, such as fish, shellfish, macroalgae, and benthic fauna, which take advantage of the complementary characteristics of their ecological niches to accomplish the recycling of nutrients and improve comprehensive farming benefits [4,5]. The snail *Bellamya purificata* is a typical benthic fauna that is used as a potential bioremediation species to improve resource utilization efficiency and purify the culture environment in IMTA owing to its feeding habits. Yang et al. found that a mixed culture of snail *Babylonia arelata*, sea cucumber *Holothuria leucospilota*, and seaweed *Gracilaria tenuistipitata* significantly promoted the growth of *B. arelata*, increased the survival rate and output, and enhanced the ecological efficiency of aquaculture [6]. *B. purificata* inhabits the silt of lakes, rivers, ditches, and ponds, and ingests organic debris and algae in its surroundings [7]. Previous studies have shown that *B. purificata* can effectively purify water and promote the circulation of materials at the sediment–water interface [8,9]. In addition, it can absorb organic debris and algae from the surrounding environment, promote degradation of organic matter in the sediment, and alleviate the accumulation of organic matter at the bottom of the pond [10].

Aquaculture ponds are complex ecosystems in which microorganisms in the sediments, animal intestinal tracts, and the waterbodies interact with each other to efficiently regulate nutrient cycling and affect aquatic animal health [11]. Microbiology can provide insight into the stability, sustainability, and dynamic variations of aquaculture ecosystems. Aquaculture activities have an obvious impact on the diversity and composition of environmental bacterial communities [12,13]. Benthic fauna activities affect biogeochemical and microbial processes in sediment [14]. With bioturbation, the structure and properties of the sediment are altered, thereby affecting the microbial community in the sediment and microbial processes, including nitrification, denitrification, and sulfate reduction [15,16]. However, the impact on microbial-driven ecological processes and functions in the culture of *B. purificata* has not been fully explained.

In the present study, high-throughput sequencing of 16S ribosomal RNA (rRNA) genes was performed to investigate the dynamic variations in benthic bacterial community composition, function, and stability caused by *B. purificata*, assess the relationships between benthic bacterial communities and environmental factors, and explore the ecological processes shaping the assembly of benthic bacterial communities. The purpose of our research was to determine the effect of *B. purificata* cultivation on the dynamics and assembly of bacterial communities in sediment. In addition, we hope that the findings will provide a theoretical reference for the optimization of IMTA and improve management strategies for *B. purificata* polyculture.

## 2. Materials and Methods

### 2.1. Culture Experiment

The experiments were performed at the Freshwater Fisheries Research Center, Chinese Academy of Fishery Sciences (120.250479° E, 31.51581° N; Wuxi, China). The experimental snail, *B. purificata*, and sediment were obtained from the Dapu Scientific Research Experiment Base of the Freshwater Fisheries Research Center (119.939129° E, 31.316981° N; Wuxi, China). These snails were fully active with an average wet weight of 2.53 ± 0.01 g. Before the experiment, they were put in a glass tank for 14 days to adapt to the laboratory conditions. The experimental sediment was the bottom silt of the aquaculture pond. The experimental sediment was dried in the sun, ground to a powder, sieved through a 100 µm mesh, and finally mixed well to ensure consistency and homogeneity. All experimental glass tanks (80 × 40 × 45 cm^3^) received a 7 cm thick layer of sediment and were then filled with filtered fresh water. The sediment in each glass tank was settled and left to stand for 14 days prior to the experiment. All snails were randomly selected and apportioned between glass tanks according to four different stocking densities: 0, 234.38, 468.75, 937.5 g/m^2^ (represented as CON, LD, MD, and HD, respectively). Each group was set up with three replicates. Throughout the experimental period, the experimental freshwater was tap water after aeration with pH of 7.14 ± 0.06 and ammonia-nitrogen of 0.09 ± 0.02 mg/L and the water temperature and dissolved oxygen were kept at 26.5 ± 0.5 °C and 6.5 ± 0.2 mg/L, respectively. Each experimental tank was aerated with the inflation pump. One-third of the water was changed every two days. The experimental lighting was indoor natural light: dark cycle. The experimental diet was commercial feed (Zhejiang Haida Feed Co., Ltd., Shaoxing, China) milled and sieved through a 100 µm mesh containing total organic matter (119.57 ± 2.31 mg/g), total nitrogen (TN) (41.02 ± 0.98 mg/g), and total phosphorus (TP) (7.07 ± 0.87 mg/g). Feeding was conducted at 16:00 daily with a feeding rate of approximately 2% of the snail’s total body weight. The experimental period lasted 80 days. All snails remained in good health and there were no mortalities.

### 2.2. Sample Collection

Sediment samples were collected at the beginning and end of the experiments. Ten sampling points were randomly selected in each glass tank and plastic tubes 2 cm in diameter were used to collect 0–1 cm surface sediment samples. All ten sediment samples from identical glass tanks were fully mixed. The sediment samples used for determining the sediment properties were dried in lyophilizer (CHRIST LYO Alpha 1-4 LD plus) at −60 °C for 96 h, ground and homogenized in a mortar, and then stored in a freezer at −80 °C for later analysis. The sediment samples used for evaluating the bacterial communities were immediately stored at −80 °C for further DNA extraction.

### 2.3. Sediment Properties Determination

The oxidation-reduction potential (ORP), pH, and dissolved oxygen (DO) in the sediment of the CON, LD, MD, and HD groups were immediately measured in situ using a portable multiparameter analyzer (Hach HQ4300 Multi/ISE/3 Channels) at the beginning and end of the experiment.

Before measuring ammonia, nitrite, nitrate, and phosphate concentrations, 1 g of sediment sample was weighed in a 50 mL centrifuge tube and shaken for 40 min with 25 mL of 2 mol/L KCl leachate, and the supernatant was collected for further analysis. The ammonia and phosphate concentrations in the sediment were analyzed using Nessler’s reagent colorimetric and molybdenum blue spectrophotometric methods, respectively [17]. Nitrate and nitrite concentrations were measured using UV spectrophotometry [18]. TN and TP concentrations were determined through the simultaneous digestion method [19] in which sediment samples (0.2 g) were dispensed into colorimetric tubes to which 25 mL alkaline potassium persulfate solution (0.074 mol/L K_2_S_2_O_4_ and 0.075 mol/L NaOH) was added, followed by dilution to 50 mL with distilled water and autoclaving at 121 °C for 30 min. TN and TP concentrations in the sediment were measured using potassium persulfate and Mo-Sb spectrophotometry [20].

### 2.4. 16S rRNA Gene Amplification and Sequencing

Bacterial DNA in the water samples was extracted using the E.Z.N.A.^®^ soil DNA Kit (Omega Bio-tek, Norcross, GA, USA) following the manufacturer’s instructions. The quality and concentration of DNA were determined using 1.0% agarose gel electrophoresis and a NanoDrop^®^ ND-2000 spectrophotometer (Thermo Scientific Inc., Waltham, MA, USA) and stored at −80 °C prior to further use.

The hypervariable region V3-V4 of the bacterial 16S ribosomal RNA gene was amplified by PCR (95 °C for 3 min, followed by 27 cycles at 95 °C for 30 s, 55 °C for 30 s, and 72 °C for 45 s with a single extension at 72 °C for 10 min, ending at 4 °C) using primers 338F (5′-ACTCCTACGGGAGGCAGCAG-3′) and 806R (5′-GGACTACHVGGGTWTCTAAT-3′). Each PCR amplification was performed in triplicate in 20 μL mixtures containing 4 μL of 5× FastPfu Buffer, 2 μL of 2.5 mM dNTPs, 0.8 μL of each primer (5 μM), 0.4 μL of FastPfu Polymerase, 10 ng of template DNA, and double-distilled H_2_O to the final volume. PCR products from the same sample were mixed and recovered using a 2% agarose gel, purified using the AxyPrep DNA Gel Extraction Kit (Axygen Biosciences, Union City, CA, USA), detected by 2% agarose gel electrophoresis and quantified by Quantus™ Fluorometer (Promega, Madison, WI, USA). A NEXTFLEX Rapid DNA Seq Kit was used to generate amplicon libraries according to the manufacturer’s instructions. Lastly, an Illumina Novaseq6000 platform was used to sequence libraries with a 250 bp paired-end strategy at Majorbio Bio-Pharm Technology Co., Ltd. (Shanghai, China).

### 2.5. Data Processing

Using an in-house Perl script to demultiplex the raw FASTQ file, quality-filtered by fastp version 0.19.6 and merged by FLASH version 1.2.7 with the following criteria: (i) The 300 bp reads were truncated at any site receiving an average quality score of <20 over a 50 bp sliding window. Truncated reads shorter than 50 bp were discarded and reads containing ambiguous characters were also discarded. (ii) Only overlapping sequences longer than 10 bp were assembled according to their overlapping sequence. The maximum mismatch ratio in the overlap region was 0.2. Reads that could not be assembled were discarded. (iii) Samples were distinguished according to the barcode and primers, and the sequence direction was adjusted, with exact barcode matching, and two nucleotide mismatches in primer matching. The optimized sequences were clustered into operational taxonomic units (OTUs) using UPARSE 7.1 with a 97% sequence similarity level. The most abundant sequence for each OTU was selected as a representative sequence. To minimize the effects of sequencing depth on alpha and beta diversity measures, the number of 16S rRNA gene sequences from each sample was rarefied to 20,000, which yielded an average Good’s coverage of 99.09%. The taxonomy of each OTU representative sequence was analyzed by RDP Classifier version 2.2 against the 16S rRNA gene database (Silva v138) using a confidence threshold of 70%.

### 2.6. Statistical Analysis

The similarity among the microbial communities in different samples was determined using principal component analysis (PCA) based on Euclidean distance. FAPROTAX software was used to establish the ecologically relevant functions of the bacterial communities. Differences in environmental factors among different groups were confirmed using a one-way ANOVA, and intergroup differences in the relative abundance of dominant phyla, families, and functional groups of the bacterial community were confirmed using the Kruskal–Wallis rank sum test.

We used redundancy analysis (RDA) to determine the contribution of environmental factors to changes in the bacterial community composition. Distance-based redundancy analysis (db-RDA) was performed using the Vegan v2.5-3 package to investigate the effects of soil physicochemical properties on soil bacterial community structure. Co-occurrence networks provide new insights into the structure of microbial communities and their construction can help identify potential biological interactions. Co-occurrence events were identified based on statistically robust correlations (|correlation coefficient| > 0.8, *p*-value < 0.05). Sloan et al. proposed a neutral community model (NCM) to quantify the importance of neutral processes in the construction of microbial communities. In this model, Nm determines the correlation between the occurrence frequency and regional relative abundance, N describes the community size, and m is the migration rate. The closer the overall fit R^2^ of the NCM is to 1, the more the process of microbial community construction is influenced by stochastic processes. The β-nearest taxon index (βNTI) can be used to identify the ecological processes governing bacterial community assembly. βNTI > 2 indicates that deterministic processes dominate and −2 < βNTI < 2 indicates that stochastic processes dominate.

All analyses were completed using the Majorbio Cloud platform (https://cloud.majorbio.com, accessed on 9 July 2022).

## 3. Results

### 3.1. Sediment Characteristics

**Figure 1:**
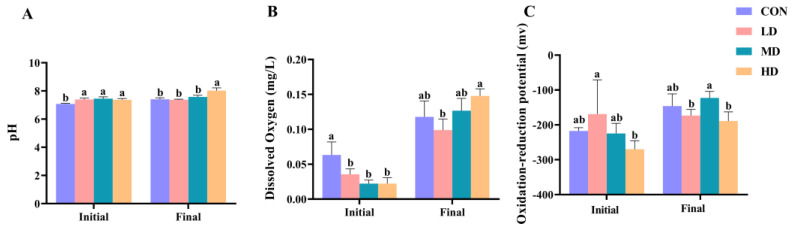
Differences in sediment characteristics of CON, LD, MD, and HD density groups, including pH (**A**), DO (**B**), and ORP (**C**); “Initial” and “Final” indicate the initial and final stages of the experimental period, respectively. Different letters for the initial and final stages indicate significant differences between CON, LD, MD, and HD density groups (*p* < 0.05). Concentrations of pH, DO, and ORP in groups marked with letter “a” were significantly higher than those in groups marked with letter “b”. Error bars are standard deviations.

Differences in pH, DO, and ORP values among the CON, LD, MD, and HD groups during the experimental period are shown in Figure 1. At the beginning of the experiment, the pH value in the CON group was significantly lower than that in the other three groups, the DO value in the CON group was significantly higher than that in the other three groups, and the ORP value in the LD group was significantly higher than that in the HD group (one-way ANOVA, *p* < 0.05). However, at the end of the experiment, the pH value in the HD group was significantly higher than that in the other three groups, the DO value in the HD group was significantly higher than that in the LD group, and the ORP values in the LD and HD groups were significantly lower than those in the MD group (one-way ANOVA, *p* < 0.05).

**Figure 2:**
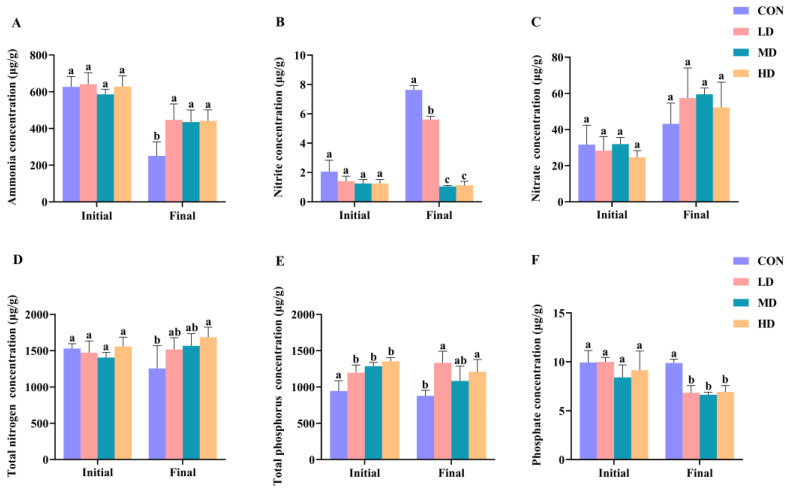
Differences in sediment N and P concentrations in CON, LD, MD, and HD density groups, including ammonia (**A**), nitrite (**B**), nitrate (**C**), TN (**D**), TP (**E**), and phosphate (**F**); “Initial” and “Final” indicate the initial and final stages of the experimental period, respectively. Different letters for the initial and final stages indicate significant differences between CON, LD, MD, and HD density groups (*p* < 0.05). Concentrations of ammonia, nitrite, nitrate, TN, TP, and phosphate in groups marked with letter “a” were significantly higher than those in groups marked with letter “b”. Error bars are standard deviations.

Differences in the ammonia, nitrite, nitrate, TN, TP, and phosphate concentrations of the sediment among the CON, LD, MD, and HD groups during the experimental period are shown in Figure 2. At the beginning of the experiment, no significant differences were observed in the ammonia, nitrite, nitrate, TN, and phosphate concentrations among the CON, LD, MD, and HD groups (one-way ANOVA, *p* > 0.05). However, TP concentrations in the CON group were significantly lower than those in the other three groups (one-way ANOVA, *p* < 0.05). When the experiment was completed, the ammonia content in the CON group was significantly lower than that in the other three groups and the nitrite concentration in the CON group was the highest, followed by that in the LD group (one-way ANOVA, *p* < 0.05). Both the TN and TP contents in the HD group were significantly higher than those in the CON group and the TP content in the LD group was also significantly higher than that in the CON group; however, the concentrations of phosphate in the CON group were significantly higher than those in the other three groups (one-way ANOVA, *p* < 0.05).

### 3.2. Bacterial Community Structure

Our study used Illumina sequencing technology based on the bacterial 16S rRNA gene to obtain 4700 distinct OTUs from the sediment samples. As shown in Figure S1, there were 250 OTUs unique to the CON group, 315 OTUs unique to the LD group, 177 OTUs unique to the MD group, 214 OTUs unique to the HD group and 1367 OTUs shared by the four groups at the beginning of the experiment. When the experiment ended, the CON, LD, MD, and HD groups contained 385, 346, 220, and 225 unique OTUs, respectively, and 1518 mutual OTUs.

**Figure 3:**
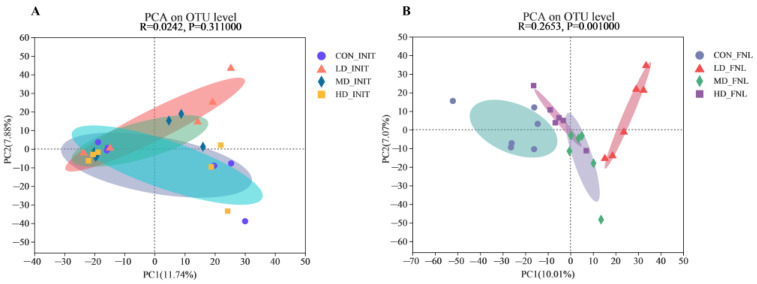
Principal component analysis of the bacterial communities at the initial (**A**) and final (**B**) stages of the experiment based on Bray–Curtis distance over all samples. CON_INIT, LD_INIT, MD_INIT, and HD_INIT represent the CON, LD, MD, and HD groups at the initial stage, respectively, and CON_FNL, LD_FNL, MD_FNL, and HD_FNL represent the CON, LD, MD, and HD groups at the final stage of the experimental period, respectively.

**Figure 4:**
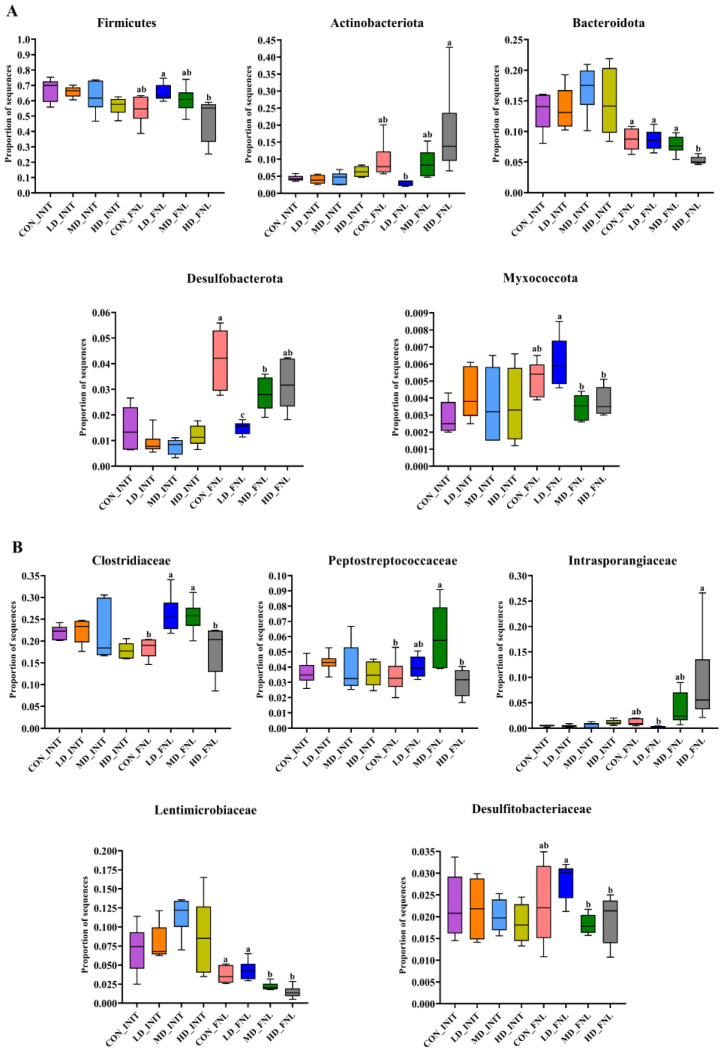
Variations in the relative abundance of dominant benthic bacterial phyla (**A**) and dominant benthic bacterial families (**B**) in CON, LD, MD, and LD groups. The box plot shows the number located at 75% of the data series, the median, and the number located at 25% of the data series in the same set of data. CON_INIT, LD_INIT, MD_INIT, and HD_INIT represent the CON, LD, MD, and HD groups at the initial stage, respectively, and CON_FNL, LD_FNL, MD_FNL, and HD_FNL represent the CON, LD, MD, and HD groups at the final stage of the experimental period, respectively. Different letters above each box in the same subfigure represent significant differences between CON, LD, MD, and HD density groups (*p* < 0.05). Relative abundances of five phyla and five families in groups marked with letter “a” were significantly higher than those in groups marked with letter “b”. Error bars are standard deviations.

PCA was applied to investigate the differences in sedimentary bacterial communities of the CON, LD, MD, and HD groups based on the Bray–Curtis distance (Figure 3). At the beginning of the experiment, PC1 and PC2 explained 11.74% and 7.88% of the total variation in sedimentary bacterial communities, respectively (Figure 4A), and at the end, 10.01% and 7.07% of the total variation in the sedimentary bacterial communities, respectively (Figure 4B). Based on the calculated *p*-values and comparison of the two images, the bacterial community showed differences at the end of the experiment and the bacterial communities of the sediment samples were categorized into four groups.

### 3.3. Bacterial Community Compositions

The dominant (10 most abundant) phyla and families in the sediment of the CON, LD, MD, and HD groups during the experimental period are shown in Figure S2. The most abundant phyla were Firmicutes, Bacteroidota, Proteobacteria, Actinobacteriota, Chloroflexi, Desulfobacterota, Halanaerobiaeota, Acidobacteriota, Myxococcota, and Patescibacteria at the beginning of the experiment (Figure S2A) and Firmicutes, Proteobacteria, Actinobacteriota, Bacteroidota, Chloroflexi, Desulfobacterota, Halanaerobiaeota, Acidobacteriota, Patescibacteria, and Myxococcota at the end of the experiment (Figure S2B).

The most abundant families in the four groups were Clostridiaceae, Caloramatoraceae, Lentimicrobiaceae, Bacillaceae, Moraxellaceae, Peptostreptococcaceae, Prolixibacteraceae, Desulfitobacteriaceae, Oxobacteraceae, and Hungateiclostridiaceae at the beginning of the experiment (Figure S2C) and Clostridiaceae, Caloramatoraceae, Peptostreptococcaceae, Moraxellaceae, Intrasporangiaceae, Lentimicrobiaceae, Bacillaceae, Desulfitobacteriaceae, Halobacteroidaceae, and Anaerolineaceae at the end of the experiment (Figure S2D).

The Kruskal–Wallis H test was used to determine differences in the dominant phyla and families between different groups (Figure 4). At the beginning of the experiment, no significant differences were observed at the phylum and family levels (Kruskal–Wallis rank sum test, *p* > 0.05). At the end of the experiment, there were five phyla and five families. The relative abundance of Firmicutes, Bacteroidota, and Myxococcota decreased with increasing density; the content of Actinobacteriota and Desulfobacterota in the HD group was significantly higher than that in the LD group (Kruskal–Wallis rank sum test, *p* < 0.05). The relative abundance of Clostridiaceae, Lentimicrobiaceae, and Desulfitobacteriaceae decreased with increasing density and the relative abundance of Intrasporangiaceae increased with increasing density; the content of Peptostreptococcaceae in the MD group was significantly higher than that in the HD group (Kruskal–Wallis rank sum test, *p* < 0.05).

### 3.4. Bacterial Functional Prediction

**Figure 5:**
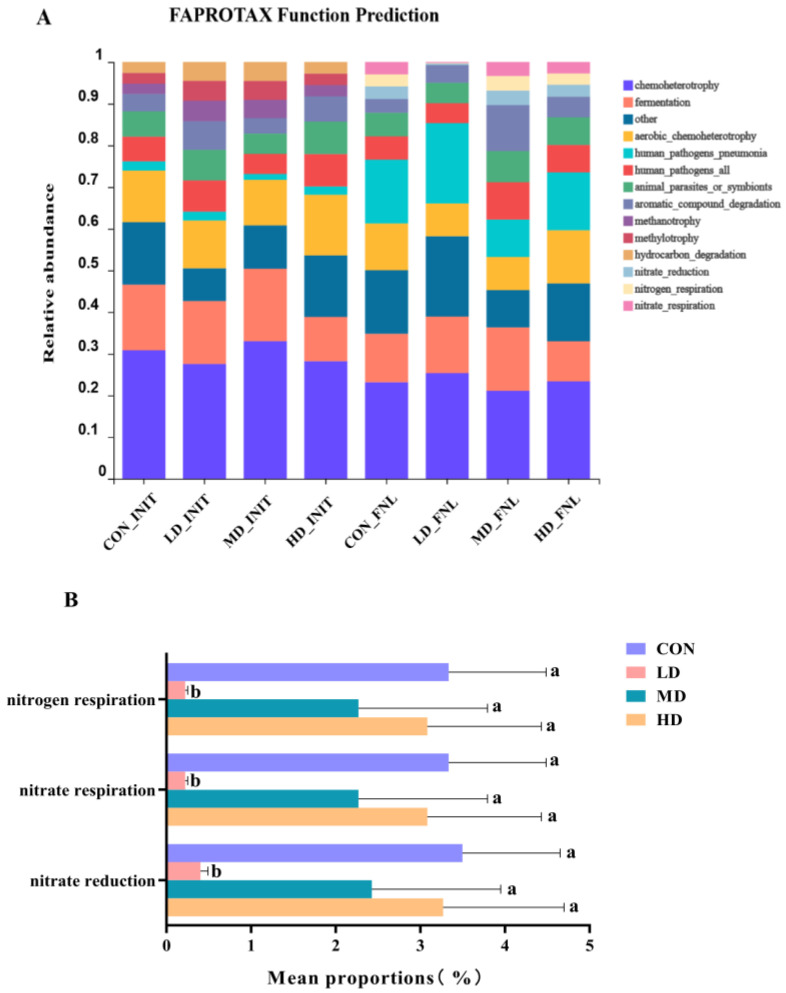
Relative abundances of the dominate functional groups ((**A**), 10 most abundant) and the significantly different functional groups ((**B**), Kruskal–Wallis H test *p* < 0.05) between CON, LD, MD, and HD density groups at the end of the experiment. CON_INIT, LD_INIT, MD_INIT, and HD_INIT represent the CON, LD, MD, and HD groups at the initial stage, respectively, and CON_FNL, LD_FNL, MD_FNL, and HD_FNL represent the CON, LD, MD, and HD groups at the final stage of the experimental period, respectively. Different letters for three functional groups indicate significant differences between CON, LD, MD, and HD density groups (*p* < 0.05). Relative abundances of nitrate reduction, nitrate respiration, and nitrogen respiration functional groups in groups marked with letter “a” were significantly higher than those in groups marked with letter “b”. Error bars are standard deviations.

A total of 55 functional groups in the CON, LD, MD, and HD groups at the end of the experiment were established using FAPROTAX. The relative abundances of the dominant functional groups (10 most abundant) and the significant differences in the dominant functional groups between density groups are shown in Figure 5. At the beginning of the experiment, the dominant functional groups were chemoheterotrophy, aerobic chemoheterotrophy, fermentation, animal parasites or symbionts, human pathogens, aromatic compound degradation, hydrocarbon degradation, methylotrophy, methanotrophy, and human pneumonia pathogen. At the end of the experiment, the dominant functional groups of the four groups were chemoheterotrophy, aerobic chemoheterotrophy, fermentation, animal parasites or symbionts, human pathogens, aromatic compound degradation, nitrate reduction, nitrate respiration, nitrogen respiration, and human pneumonia pathogen. There were significant differences in nitrate reduction, nitrate respiration, and nitrogen respiration functional groups among the four density groups at the end of the experiment (Figure 5B) and the mean proportions of these three functional groups in the LD group were significantly lower than those in the other three density groups (Kruskal–Wallis rank sum test, *p* < 0.05).

### 3.5. Effect of Environmental Factors on Bacterial Abundance

**Figure 6:**
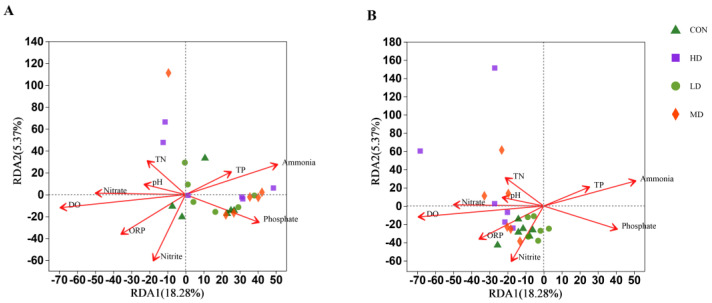
Redundancy analysis (RDA) for the relationship between the bacterial operational taxonomic units (OTUs) of sediment and nutrients in CON, LD, MD, and LD density groups at the (**A**) initial and (**B**) final stages of the culture cycle.

According to the OTU clustering results and the OTU distribution of each sample, redundancy analysis (RDA) was carried out in combination with nitrogen and phosphorus nutrients in the sediment. The results are shown in Figure 6. At the end of the experiment: ammonia, phosphorus, and TP were negatively correlated with bacterial community composition in four density groups; DO, ORP, nitrite, and nitrate were positively correlated with the bacterial community composition in four density groups; and TN and pH were positively correlated with bacterial community composition in MD and HD groups.

### 3.6. Co-Occurrence Patterns of Bacterial Taxa during the Culture Cycle

**Figure 7:**
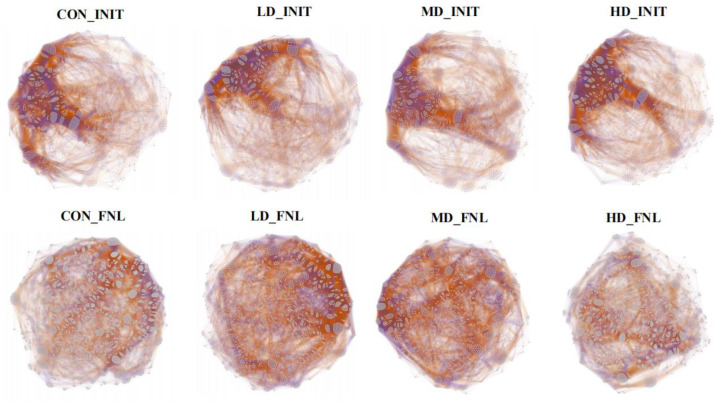
Co-occurrence networks of bacterial communities in CON, LD, MD, and LD density groups during the culture cycle. CON_INIT, LD_INIT, MD_INIT, and HD_INIT represent the CON, LD, MD, and HD groups at the initial stage, and CON_FNL, LD_FNL, MD_FNL, and HD_FNL represent the CON, LD, MD, and HD groups at the final stage of the experimental period.

The co-occurrence network of bacteria showed that the community structure in the final period of the experiment was significantly more complex than at the beginning of the experiment (Figure 7). At the beginning of the experiment, the co-occurrence network of the four density groups consisted of 1462 nodes with 117,327 edges, 1469 nodes with 107,979 edges, 1392 nodes with 97,268 edges, and 1513 nodes with 125,572 edges (Table 1). At the end of the experiment, the co-occurrence network of the CON, LD, MD, and HD groups consisted of 1961 nodes with 145,023 edges, 1859 nodes with 137,972 edges, 1802 nodes with 126,824 edges, and 1829 nodes with 131,918 edges, respectively (Table 1). Additionally, we compared the ratio of positive to negative edges in the network between the two stages. At the end of the experiment, the positive edge ratio increased with the increasing density and ranged from the 59.24% in the LD group to 68.89% in the HD group. The negative edge ratio decreased from 40.76% in the LD group to 31.11% in the HD group as density increased. The ratio of positive to negative edges in the network is increasing with increasing density.

### 3.7. Mechanisms Involved in Regulating Bacterial Community Structure

The NCM can be used to observe the distribution of species abundance in the sediment bacterial community. Approximately 60–70% of the community variation in bacterial communities during the beginning and final experiments can be explained by NCM (Figure S3). The results suggested that stochastic processes influenced the shaping of sediment bacterial communities in different density groups. The Nm value of the different density groups at the end was higher than that at the beginning, indicating a higher species dispersal ability. Thus, the NCM results suggested that the species dispersion of the sediment bacterial community was higher in the final stage than at the beginning of the experiment. In addition, the median values of βNTI at the beginning and end of the experiment ranged from −1.131 to 10.843, and the mean βNTI for all experimental groups in all culture phases ranged between −2 and 2 (Figure S4). This supported the conclusion that stochastic processes contributed the most to the compositional structure of the sediment bacterial community in the four groups.

## 4. Discussion

### 4.1. Bacterial Community Compositions and Functions Affected by B. purificata Cultivation

Aquaculture activities can have an obvious impact on the diversity and composition of the bacterial community during the culture process [12,13]. Aquatic animal farming and daily management operations, including feed input and bioturbation, can cause several environmental changes, thus influencing bacterial communities [21,22]. Sun et al. found that *Bellamya aeruginosa* could promote the growth of benthic microorganisms and induce variations in microbial community composition through bioturbation [23]. In the present study, differences in bacterial community composition among the four groups also indicated a significant effect of *B. purificata* cultivation on bacterial communities in the sediment. At the end of the experiment, *B. purificata* cultivation at high stocking densities had significantly decreased the relative abundance of phylum Bacteroidota in the sediment and *B. purificata* cultivation at low stocking densities had significantly decreased the relative abundance of phylum Desulfobacterota in the sediment. Meanwhile, the relative abundance of the phylum Actinobacteriota significantly increased with increasing stocking density. Bacteroidota are typically anaerobic bacteria that play a role in organic matter circulation by decomposing a wide variety of organic compounds [24,25]. Desulfobacterota is a sulfate-reducing bacterium that transports electrons from hydrogen sulfide to nitrate or nitrite and mediates the sulfur cycle in the environment. Actinobacteriota are aerobic bacteria that contribute to maintaining the stability of pond ecosystems by decomposing many organic and inorganic materials, promoting material circulation, energy flow, and the formation of soil agglomerate structures [26]. Hence, *B. purificata* cultivation could significantly alter the microbial ability to degrade organic matter and affect the sulfur cycle mediated by bacteria. In particular, a high stocking density could inhibit anaerobic decomposition and improve aerobic decomposition in the organic matter, thereby helping to stabilizing the aquaculture ecosystem. *B. purificata* cultivation at low stocking densities weakened microbial sulfate-reducing ability.

In addition, *B. purificata* cultivation at low stocking densities also had a significant impact on the functional groups of the bacterial community. The results of the FAPROTAX function prediction in the present study showed that three functional groups related to the nitrogen cycle, namely nitrate reduction, nitrogen respiration, and nitrate respiration, were significantly decreased in the LD group. Nitrate reduction is an allosteric process that uses organic matter as an electron donor to reduce NO_3_^−^ to NH_4_^+^ and re-enter the environment to participate in the nitrogen cycle [27]. Nitrate respiration can be divided into three categories: denitrification, dissimilatory reduction of nitrate to ammonium (DNRA), and the anammox process with nitrate as an indirect electron acceptor [28]. Nitrate respiration in soil ecosystems mainly focuses on reducing reactive nitrogen loss and improving soil nitrogen retention capacity [29,30]. Significantly decreased nitrate reduction, nitrogen respiration, and nitrate respiration in the LD group indicated that *B. purificata* cultivation at low stocking densities could inhibit the denitrification process, especially the process of reducing NO_3_^−^ to NH_4_^+^. Previous studies have reported an important effect of benthic fauna on nitrogen reactions in sediment [31,32]. Fang et al. found that polychaete bioturbation decreased nitrogen and nitrate respiration by bacterial functional groups in sediment, which was consistent with our results [33]. However, this effect of *B. purificata* cultivation on nitrogen reactions in the sediment disappeared with increasing stocking density.

### 4.2. Bacterial Co-Occurrence Networks Affected by B. purificata Cultivation

Most bacteria thrive in multitudinous communities rather than living in isolation and the close interactions developed can bring more benefits to the whole community and shape its structure and function [34,35]. Bacterial co-occurrence networks have been widely developed and used to explore and evaluate complex bacterial structures and their interconnected patterns [35]. Significant variations in the co-occurrence networks of bacterial communities have been observed in many aquaculture ecosystems as culture progresses [36,37,38]. In the present study, the number of nodes and edges in co-occurrence networks of sediment bacterial communities at the end of the experiment were much higher than those at the initial stages, which suggested a closer interaction between bacterial communities and increased resistance to disturbance [39]. More complex bacterial communities were revealed by increased nodes and edges in the co-occurrence networks of sediment bacterial communities after the experimental period. At the end of the experiment, the positive edge ratio in the bacterial co-occurrence networks showed an increasing trend with increasing stocking density, whereas the negative edge ratio decreased with increasing stocking density. The negative edge ratio in the present study ranged from 40.76% in the LD group to 31.11% in the HD group, which was similar to the 32.8 to 39.7% negative edge ratio found in the bacterial co-occurrence networks for sediments [35]. Positive interactions (such as mutualism) drive bacterial community evolution by enhancing biological fitness [40]. Negative interactions (such as competition, parasitism, and predation) that probably originate from a wide range of co-exclusion mechanisms, including direct competition, toxin production, environmental modification, differential niche adaptation, and driving evolution by selective pressure [34,41]. Low negative edge ratios generally reflect low intensities of competition or niche differentiation and indicate prevalent collaboration or niche sharing, which can result from heterogeneous microenvironments by reducing direct competition [35]. Benthic fauna can effectively improve the heterogeneity of the sediment environment through bioturbation. *B. purificata*, as a typical benthic animal, can create a heterogeneous sediment environment, thus decreasing direct competition and promoting collaboration or niche sharing in bacterial communities. In addition, more feed inputs also tend to imply higher chance and degree of heterogeneity of the sediment. However, our study aimed at evaluating the whole effects of *B. purificata* cultivation at different stocking densities on the bacterial communities in sediment. We considered these two effects, namely *B. purificata* bioturbation and artificial feed inputs as a whole. Our study was not intended to distinguish and assess the separate impact of *B. purificata* bioturbation and artificial feed inputs. Hence, the effects of *B. purificata* cultivation on the bacterial co-occurrence networks might be the combined result of *B. purificata* bioturbation and feed inputs. The higher the stocking density, the stronger the effect.

### 4.3. Environmental Factors and Their Correlations with Bacterial Community Affected by B. purificata Cultivation

Environmental factors in aquatic ecosystems directly affect the composition and distribution of bacterial communities [42,43]. In the present study, *B. purificata* cultivation significantly changed the pH, DO, TN, TP, nitrite, ammonia, and phosphate concentrations in the sediment. Normally, there is a certain correlation between structural changes in bacterial communities and the environmental factors in which they live [44,45]. RDA analysis showed that DO, pH, and nutrient content are critical environmental factors influencing the composition of bacterial communities, which was consistent with the results of previous studies [4,46,47]. Previous studies have shown that dissolved oxygen and nutrient bioavailability can be the main factors causing considerable variations in the composition and function of bacterial communities, especially in small environments [48,49,50]. Meanwhile, the pH range of 7.5–8.0 in aquaculture pond sediment has high microbial activity and is conducive to the decomposition of organic matter and hastens the process of nutrient recirculation [51]. Macrobenthos bioturbation can affect the pH value and transport oxygen from water bodies to sediments [52,53,54,55]. Increasing stocking density in *B. purificata* cultivation resulted in a stronger intensity of bioturbation. The highest pH and DO values in the HD group might indicate that *B. purificata* cultivation at a high stocking density could alter the bacterial community by influencing the pH and DO with the strongest intensity of bioturbation. In addition, Kolukirik et al. found that TP and TN were factors restricting bacterial growth and bacterial abundance and activity depended on their content [56]. The TN and TP contents were significantly higher in the HD group than in the CON group and *B. purificata* cultivation decreased the nitrite and phosphate contents while increasing the ammonia content. These findings revealed obvious impacts of *B. purificata* on the nutrient cycles, thus affecting the bacterial community structure, which was consistent with previous research results [57,58].

### 4.4. Bacterial Community Assembly Affected by B. purificata Cultivation

Our results showed that, according to the fit of the neutral model, the stochastic process of the bacterial community structure overrides the deterministic process. In particular, stochastic processes in the bacterial community structure in all groups became increasingly dominant as the experiment progressed. However, the neutral models (Rsqr) were similar between the different groups at the end of the experiment, which implied that *B. purificata* cultivation had limited impacts on the bacterial community assembly and might not be strong enough to change environmental conditions for selecting specific microorganisms. Similar results have been observed in both natural and artificial aquaculture ecosystems. Stochastic processes were dominant in governing the assembly of bacterial communities in aquaculture ponds, lakes, and other recirculating aquaculture systems [36,37,59,60,61]; correspondingly, aquaculture activities in these regions had very limited effects on bacterial community assembly. Zeng et al. found that seasonality overwhelmed aquaculture activity in determining the bacterial community assembly in Taihu Lake [59]. Hou et al. found that disturbance by apex predator mandarin fish had only slight effects in shaping bacterioplankton communities in crustacean aquaculture ponds [37]. In the present study, the slight effects of *B. purificata* cultivation on bacterial community assembly were reflected in the highest migration rate in the LD group and the lowest migration rate in the HD group, which indicated a potential impact of *B. purificata* cultivation on species dispersal ability [36], which would be promoted at low stocking density and inhibited at high stocking density.

## 5. Conclusions

The present study has demonstrated that the cultivation of *B. purificata* induced changes in the composition and function of the bacterial community. By comparing with other density groups, it reduced the decomposition of anaerobic organic matter and increased the decomposition of aerobic organic matter at high stocking density, while at low stocking density it reduced the number of bacteria involved in sulfate reduction and inhibited the denitrification process to some extent. *B. purificata* could decrease direct competition and promote collaboration or niche sharing in bacterial communities through creating a heterogeneous sediment environment, especially at the high stocking density. Moreover, *B. purificata* cultivation resulted in greater changes in the environmental factors. Variations in DO, pH, TN, nitrate, and nitrite levels were closely related to the altered composition and function of the bacterial communities. Stochastic processes dominated the bacterial community assembly in the sediment and *B. purificata* cultivation had limited impacts on the bacterial community assembly. The findings of this study provide a greater understanding of the effect of *B. purificata* cultivation on bacterial community composition and assembly. Meanwhile, it was worth noting that we set the *B. purificata* cultivation as the subjects and we considered *B. purificata* bioturbation and artificial feed inputs as a whole in the present study. We aimed at evaluating the whole effects of *B. purificata* cultivation at different stocking densities on the bacterial communities in sediment rather than intending to distinguish and assess the separate impact of *B. purificata* bioturbation and artificial feed inputs. Distinguishing and evaluating the separate effect of *B. purificata* bioturbation and artificial feed inputs on bacterial communities in sediment will be our important research direction in the future.

## Figures and Tables

**Table 1:** Topological parameters of co-occurrence networks based on bacterial communities in different density groups during the culture cycle.

	Initial				Final			
	CON	LD	MD	HD	CON	LD	MD	HD
Nodes	1462	1469	1392	1513	1961	1859	1809	1829
Edges	117,327	107,979	97,268	127,572	145,023	137,972	126,824	131,918
Positive edge ratio	65.92%	63.16%	60.55%	63.90%	59.99%	59.24%	65.17%	68.89%
Negative edge ratio	34.08%	36.84%	39.45%	36.10%	40.01%	40.76%	34.83%	31.11%

## Data Availability

The datasets presented in this study can be found in online repositories. The names of the repository/repositories and accession number(s) are PRJNA896351.

## Supplementary Materials

The following supporting information are available online at https://www.mdpi.com/article/10.3390/biom13020254/s1. Figure S1: Venn diagrams of CON, LD, MD, and HD density groups at operational taxonomic unit (OUT) level. Figure S2: Relative abundances of the dominate phylum (10 most abundant) and family (10 most abundant) in CON, LD, MD, and HD density groups at the initial and final stages of the experiment. Figure S3: Fit of the neutral community model (NCM) for bacterial communities in four density groups at the initial and final stages of the culture cycle. Figure S4: Distribution of the β-nearest taxon index (βNTI) of the bacterial communities in the sediment of four density groups at the initial and final stages of the culture cycle.
