# Supplementary material for: Effects of Bellamya purificata Cultivation at Different Stocking Densities on the Dynamics and Assembly of Bacterial Communities in Sediment
Source: Biomolecules. 2023 Jan 30;13(2):254. doi: 10.3390/biom13020254 (PMC9953186; doi:10.3390/biom13020254)
Supplement: Supplementary file 1 [file biomolecules-13-00254-s001.zip › biomolecules-2037585-supplementary.pdf]

Supplemental material

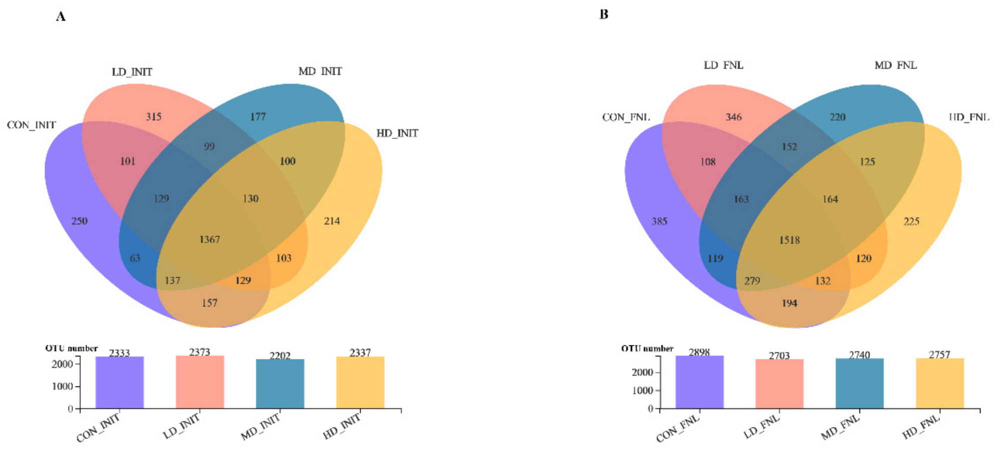

Figure S1. Venn diagrams of CON, LD, MD, and HD density groups at operational taxonomic unit (OTU) level, including at (A) the initial stages and (B) the final stages of the experiment. The Y axis of the bar chart represents the number of OTUs. CON_INIT, LD_INIT, MD_INIT, and HD_INIT represent the CON, LD, MD, and HD groups at the initial stage, and CON_FNL, LD_FNL, MD_FNL, and HD_FNL represent the CON, LD, MD, and HD groups at the final stage of the experimental period.

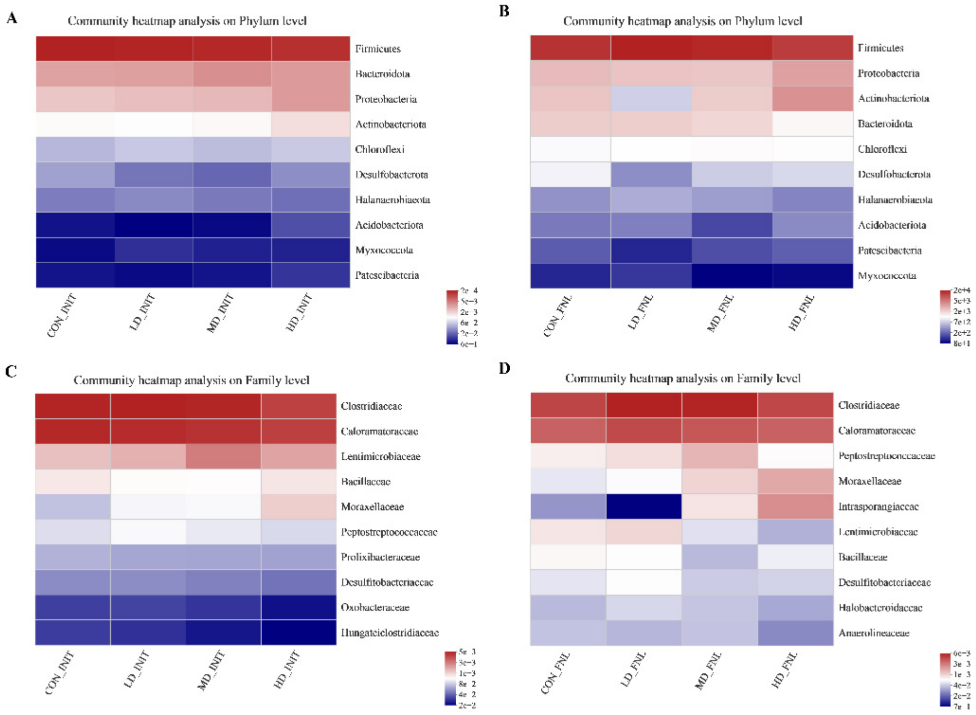

Figure S2. Relative abundances of the dominate phylum (10 most abundant) and family (10 most abundant) in CON, LD, MD, and HD density groups at the initial and final stages of the experiment. CON_INIT, LD_INIT, MD_INIT, and HD_INIT represent the CON, LD, MD, and HD groups at the initial stage, and CON_FNL, LD_FNL, MD_FNL, and HD_FNL represent the CON, LD, MD, and HD groups at the final stage of the experimental period.

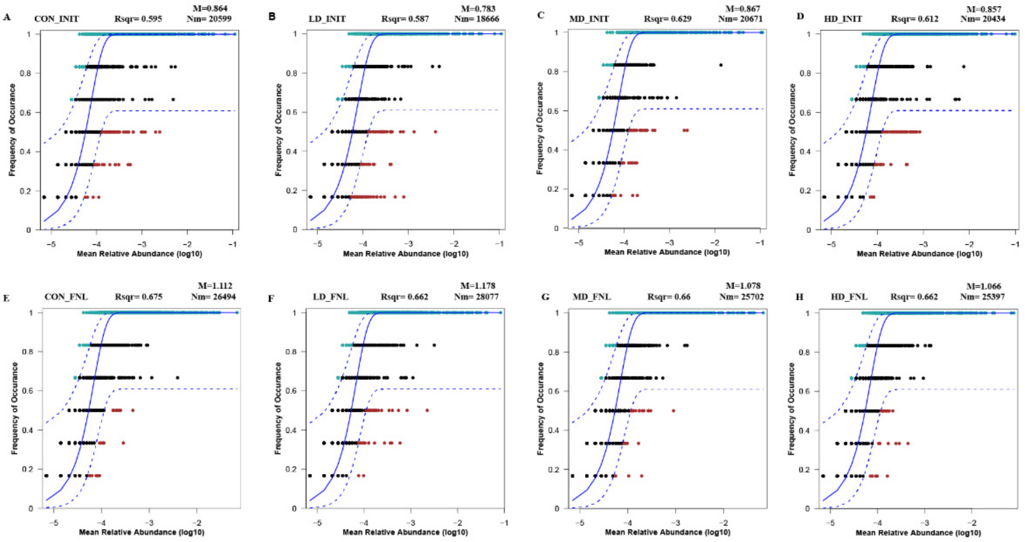

Figure S3. Fit of the neutral community model (NCM) for bacterial communities in four density groups at the initial and final stages of the culture cycle. The solid and dashed lines indicate the best fit to the NCM and 95% confidence intervals around the model prediction, respectively. Nm indicates the meta-community size × immigration, and Rsqr indicates the fit to this model. CON_INIT, LD_INIT, MD_INIT, and HD_INIT represent the CON, LD, MD, and HD groups at the initial stage, and CON_FNL, LD_FNL, MD_FNL, and HD_FNL represent the CON, LD, MD, and HD groups at the final stage of the experimental period.

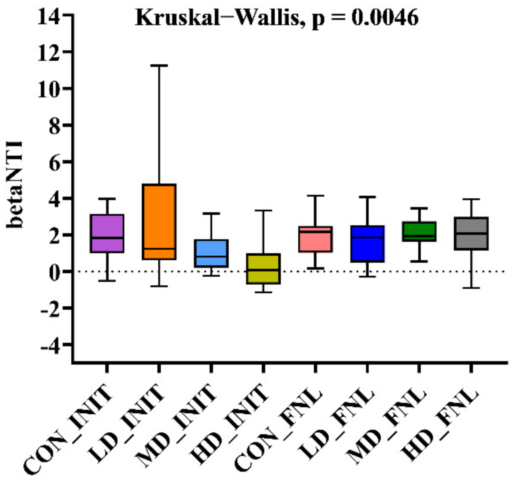

Figure S4. Distribution of the β-nearest taxon index (βNTI) of the bacterial communities in the sediment of four density groups at the initial and final stages of the culture cycle. The box plot shows the number located at 75% of the data series, the median and the number located at 25% of the data series in the same set of data. CON_INIT, LD_INIT, MD_INIT, and HD_INIT represent the CON, LD, MD, and HD groups at the initial stage, and CON_FNL, LD_FNL, MD_FNL, and HD_FNL represent the CON, LD, MD, and HD groups at the final stage of the experimental period. Error bars are standard deviations.
